# Vacuum assisted closure technique: a short review

**DOI:** 10.11604/pamj.2017.28.246.9606

**Published:** 2017-11-21

**Authors:** Sankalp Yadav, Gautam Rawal, Mudit Baxi

**Affiliations:** 1 Department of Medicine & TB, Chest Clinic Moti Nagar, North Delhi Municipal Corporation, New Delhi, India; 2 Respiratory Intensive Care, Max Super Specialty Hospital, Saket, New Delhi, India; 3 Department of Orthopedics, Sri Aurobindo Medical College and Postgraduate Institute, Indore, Madhya Pradesh, India

**Keywords:** Negative pressure, negative pressure wound device, VAC, vacuum therapy, vacuum sealing, wounds, wound closure, wound healing

## Abstract

The management of difficult to heal wounds has always been a cause of concern for the treating clinicians. There has been a tremendous increase in the number patients presenting with difficult to heal wounds. The conventional techniques have been in use since the long time for the management of these wounds, yet desired results are not achieved always. Thus a newer novel technique which might be useful in the difficult to heal wounds and delivering at par or better results as compared to the conventional techniques is the need of the hour.

## Commentary

A wound is defined as damage or disruption to the normal anatomical structure and function [1]. This can range from a simple break in the epithelial integrity of the skin or it can be deeper, extending into subcutaneous tissue with damage to other structures such as tendons, muscles, vessels, nerves, parenchymal organs and even bone [2]. Wound healing remains a challenging clinical problem and correct, efficient wound management is essential [3]. The primary aim of the practicing clinicians is to achieve a high rate of success in the wound healing. In the countries like India, where the chances of wound infection are high due to multiple issues, the importance of novel techniques of wound care are essential. The scientific community has always looked for a new and more effective wound care techniques, particularly with an emphasis on new therapeutic approaches and the development of technologies for acute and chronic wound management [3]. So far very few remarkable achievements have been reported in the scientific literature. Despite numerous advances, chronic and other difficult to manage wounds continue to be a challenge for the clinicians [4]. The relatively newer techniques like negative pressure wound therapy (NPWT) using the vacuum assisted closure (VAC) are very promising and are also useful in the management of difficult to heal wounds. In this short review, we highlight the importance of the negative pressure wound device (NPWD) in the successful wound healing. We will also discuss the importance of this technique in a developing country like India. The practice of exposing a wound to sub-atmospheric pressure is relatively new and was first described by Fleischmann et al. in the year 1993, who first reported the use of sub-atmospheric pressure for an extended period to promote debridement and healing following the successful use of this technique in 15 patients with open fractures [5]. Their study reported that the treatment method of reducing the pressure inside the wound was very effective. However, the first reports about the use of negative pressure wound device came from Argenta and Morykwas in the year 1997 [4]. The use of controlled levels of negative pressure application has been shown to accelerate debridement and promote healing in various types of wounds [6]. This optimum level of negative pressure appears to be around 125 mmHg below ambient and there is evidence that this is most effective if applied in a cyclical fashion of five minutes on and two minutes off [6]. Earlier studies used more conventional methods such as a wall suction apparatus or surgical vacuum bottles for creating the negative pressure [6]. However, there were multiple problems present in the use of these conventional methods [7]. In the year 1995, a commercial system for promoting vacuum assisted closure (VAC) also known as vacuum therapy, vacuum sealing or topical negative pressure therapy, was introduced into the United States market [6]. This equipment, called the VAC, was designed to overcome some of the problems associated with conventional methods for the creation of negative pressure. The heart of the system is a microprocessor-controlled vacuum unit that is capable of providing controlled levels of continuous or intermittent sub-atmospheric pressure ranging from 25 to 200 mmHg [6]. Later on a number of improvements were made to this basic model of VAC.

### The mechanism of action

The first attempt to explain the physiological basis of the observed good clinical results using NPWT was made by the Morykwas et al. 1997 [8]. Morykwas and colleagues postulated that multiple mechanisms might be responsible for the beneficial results obtained from VAC [6]. They suggested that removal of interstitial fluid decreases localized edema and increases blood flow, which in turn decreases tissue bacterial levels. It has since been proposed that the application of sub-atmospheric pressure produces mechanical deformation or stress within the tissue resulting in protein and matrix molecule synthesis and enhanced angiogenesis [9, 10]. The VAC treatment applies localized negative pressure applied to a special dressing positioned within the wound cavity or over a flap or graft that assists with the removal of interstitial fluid thereby decreasing localized edema and increasing the blood flow [6]. Thus, decreasing the tissue bacterial levels. Also, the mechanical deformation of cells increases the rate of cell proliferation due to protein and matrix molecule synthesis [6]. The technique has yielded good results in the studies reported, and also this technique is comparatively cost effective as compared to the conventional treatments, particularly in wounds that are difficult to heal [6].

### Clinical evidences

Negative pressure wound therapy using the VAC is one of the most important modes of treatment used in modern wound management [11-15]. A number of clinical studies, both on animals and humans have been done so far. Most of these studies have reported the use of VAC as at par and at times better with the conventional wound closure techniques [16]. Argenta and Morykwas in the year 1997 reported the results of their study on 300 human subjects of which 296 patients responded favorably to the VAC [4]. Similarly, numerous other papers have described the use of VAC in the treatment of a variety of wound types including soft tissue injuries prior to surgical closure [17], extensive degloving injuries [18,19], various grafting or reconstructive surgery [20] and infected sternotomy wounds [21-23]. Smith et al. 1997, in a retrospective review of open abdomen management and temporary abdominal closure, suggested the use of VAC as the treatment method of choice [24]. Vikatmaa et al. 2008, studied 14 RCTs and reported that in all trials, NPWT was at least as effective, and in some cases, more effective than the control treatment [25]. The same has been reported from other parts of the globe [26]. VAC has also been used in the treatment of donor sites, especially in areas that are difficult to manage by using conventional techniques [27] such as those of the radial forearm [28]. Andrabi et al. 2007, showed that usage of the VAC in the closure of laparotomy wound was much superior and quicker than conventional methodology [29]. VAC has also been used in conjunction with split thickness skin grafts in the treatment of burns and is claimed to be particularly useful for body sites with irregular or deep contours such as the perineum, hand or axilla [30-32]. Numerous case histories have described the successful use of VAC in a variety of non-healing or chronic wounds. These include a recalcitrant below knee amputation wound pressure sores [33-38] and a suspected Brown Recluse Spider bite [39], leg ulcers, and a group of 30 patients with longstanding wounds that were deemed unsuitable for reconstructive surgery, 26 of whom responded favorably to the treatment [40].

### The method

The VAC involves a six step method. The following are the VAC steps, as detailed by Thomas in 2001 [6]: the foam dressing is cut to the approximate size of the wound with scissors and placed gently into position→ The perforated drain tube is then located on top of the foam and a second piece of foam placed over the top. For shallower wounds, a single piece of foam may be used and the drainage tube is inserted inside it→ The foam, together with the first few inches of the drainage tube and the surrounding area of healthy skin, is then covered with the adhesive transparent membrane supplied. At this stage it is important to ensure that the membrane forms a good seal both with the skin and the drainage tube →. The distal end of the drain is connected to the VAC unit, which is programmed to produce the required level of pressure→ Once the vacuum is switched on, the air is sucked out of the foam causing it to collapse inwards drawing the edges of the wound in with it→ Fluid within the wound is taken up by the foam and transported into the disposable container within the main vacuum unit [6].

### Cost of treatment

Greer et al. 1999, discussed some of the practical problems associated with the application of the VAC system [37]. Although the technique appears to be very costly since it involves the expenditure on the purchase cost or hire charges of the VAC machine itself, besides it is necessary to purchase disposable foam dressings and drainage tubes, canisters and adhesive drapes, which together could easily cost in excess of 25 per day [6]. Yet the reports about the cost effectiveness of VAC are available in the scientific literature [41]. One reason could well be the time duration for the healing of the wounds which is shorter in the VAC as compared to the conventional methods. Moues et al. 2005, showed that NPWT had significantly higher material expenses (p < 0.001), but significantly lower nursing expenses (p = 0.043) [42]. Still further studies investigating the cost-effectiveness of VAC therapy compared with conventional methods of wound management are required in future.

### The Indian perspective

The low income countries are having a number of issues related to public health [43]. The use of NPWT in the Indian studies has not been reported extensively. Only few Indian studies have provided insights into the NPWT use in Indian setting [16,44-46]. The NPWT using the VAC is definitely having an advantage in countries like India, where the patient load on the health centers is very high [47,48]. In countries like India, where more than 40% of the population earns less than one US dollar per day and where only a small portion of government budget goes to health there is an urgent need of faster and cheaper wound healing techniques [48-53]. The health care information about the newer and cheaper techniques will help a great deal in the exponentially growing population [47]. In such a scenario any wound healing technique that will work faster than the conventional techniques and deliver at par or at times better results is definitely a boon [16]. Hussain et al. 2012, reported that the VAC has certain advantages like it is easy to handle, hospital admission is not essential, good patient compliance and satisfaction, require minimal training to maintain vacuum at home, can be applied to multiple cases at the same time and give adequate mobility to the patient [16]. The VAC will also reduce the total time spent in the hospital and this is ideal for already overloaded hospitals [47]. Besides, the number of follow-ups will also be reduced in cases involving the VAC. But the situation in the rural areas is graver [54]. As, the VAC is not available everywhere in the developing countries and the usage of VAC in the rural areas is very difficult due to difficulties related to terrain, availability of devices, cost issues, etc. [46]. Thus the use of indigenous substitutes of the wound management which can be used in the rural areas of developing countries is essential [46]. Further the advantages and disadvantages of VAC are summarized in Table 1, as explained by Thomas in the year 2001 [6]. In the absence of larger studies with adequate sample size from various population groups the results available so far from the published studies suggest that the VAC is a promising technique. Further research will help in analyzing the cost effectiveness of the VAC over the conventional techniques. But till that time, the data available from scientific literature suggest VAC to be a cost effective technique, resulting in at par or at times better wound healing, with few serious complications.

**Table 1:** Indications and contraindications for the use of VAC [6]

**The principal indications for the use of the mains powered VAC are**
Acute and traumatic wounds
Sub-acute wounds (i.e. dehisced incisions)
Chronic open wounds (stasis ulcers and diabetic ulcers)
Flaps
Meshed graft
Pressure ulcers
**The small ambulant unit is recommended for:**
Venous stasis ulcers
Pressure ulcers
Lower extremity diabetic ulcers
Lower extremity flaps
Dehisced incisions
Grafts
**Contraindications for both systems include:**
Fistulas to organs or body cavities
Necrotic tissue in eschar
Untreated osteomyelitis
Malignancy in the wound

## Competing interests

The authors declare no competing interests.
